# Spatial-Temporal Coupling Analysis of the Coordination between Urbanization and Water Ecosystem in the Yangtze River Economic Belt

**DOI:** 10.3390/ijerph16193757

**Published:** 2019-10-06

**Authors:** Han Han, Huimin Li, Kaize Zhang

**Affiliations:** 1 Business School, Hohai University, Nanjing 211100, China; 170212070002@hhu.edu.cn; 2 Department of Construction Engineering and Management, North China University of Water Resources and Electric Power, Zhengzhou 450046, China; 3 Henan Key Laboratory of Water Environment Simulation and Treatment, Zhengzhou 450045, China

**Keywords:** urbanization, water ecosystem, coupling coordination degree, Yangtze River Economic Belt

## Abstract

As a primary pioneering region in China’s ongoing urbanization process, the Yangtze River Economic Belt’s (YREB’s) urbanization process is itself continually accelerating, causing increasing pressure on the area’s water ecosystem. It is necessary to examine the coordination relationship between the urbanization system and the water ecosystem in the YREB for realizing sustainable urban development. To this purpose, we use two comprehensive index systems, along with an improved coupling coordination degree (CCD) model. This method is used to analyze the coordination between urbanization and the water ecosystem across spatial gradients and temporal scales in the YREB, from 2008 to 2017. The factors acting as obstacles were diagnosed by utilizing the obstacle degree model. The results show that: (1) the coordination state of each region gradually improved during the 2008–2017 period. In terms of spatial distribution, the coordination state between two systems gradually increased from east to west. Moreover, the spatial differences across the 11 analyzed regions gradually narrowed with the passage of time. (2) The coordination between the two systems, from 2008 to 2017, evolved from a state of serious imbalance to a state of good coordination. The two systems passed from an initial period of imbalance or antagonism, coupled with rapid growth (2008–2011), through a period of basic coordination with steady growth (2011–2014), and finally toward a period of good coordination with slow growth (2014–2017). (3) Spatial urbanization and pressures on subsystems are the key factors acting as obstacles in the urbanization system and water ecosystem, respectively. Facing the process of rapid urbanization in China, the coupling analysis of the coordination between urbanization and the water ecosystem can help the government to formulate a reasonable new-type urban development strategy. This strategy will play an important role in China’s sustainable urban development and water environmental protection. The findings of this study provide important support for urban planning in the future.

## 1. Introduction

Water is essential to human survival and development. Water is also an important resource for the continuous evolution of urban systems to higher levels in the urbanization process [1]. However, the continuous acceleration of the urbanization process usually results in water shortages, water pollution, and damage to the water ecosystem [2,3,4]. Urban expansion is taking place globally. The global urban population has grown from 51 million in 1950 to 4.2 billion in 2018 [5]. This expansion is particularly prominent in developing countries [6]. Relevant statistical data show that in the past 40 years, China, India, and Africa have experienced a rapid urbanization process [7]. According to the China Statistical Yearbook, since the economic reform of 1978, China has experienced rapid urbanization [8], rising from 19.39% in 1980 to 58.52% in 2017. In India, the percentage of the urban population has increased from 17% in 1951 to approximately 35% in 2017 [9]. Future urban growth is likely to continue. It is estimated that by 2050, China will have an additional 250 million urban residents, while India’s urban population will increase by approximately 400 million, and Nigeria by approximately 190 million [6]. Urban expansion in the peripheral areas of major city centers generally takes place at the expense of prime farmlands [10]. This accelerated urbanization process requires a considerable amount of both water and land resources, thereby resulting in water shortages and damage to the water recycling system [11]. These pressures will ultimately threaten the areas’ ability to realize sustainable urban development [12].

The water ecosystem is an organic whole composed of organisms and a water environment in a specific physical space. In this space, organisms and the water environment interact and restrict each other, and they are in a dynamically balanced state [13,14]. A healthy natural water ecosystem can provide human beings with water services functions that will help ensure sustainable socio-economic development [15]. However, according to a recent survey [16], more than half of China’s cities currently face water problems, including water shortages, water eutrophication, and water pollution. The contradiction between the protection of the water ecosystem and the ongoing development of urbanization is becoming increasingly prominent. Hence, there is a clear need to balance the relationship between urban development and protecting water resources. In this respect, it is important to fully understand the coordination between urbanization and the water ecosystem, as this understanding can provide the basis of a management framework for the future sustainable development of these two systems. 

At the end of the 20th century, environmental issues caused by urbanization, such as water pollution, atmospheric pollution, and soil damage, have attracted widespread attention from scholars [17,18]. Various scholars have applied the Environmental Kuznets Curve (EKC) hypothesis to explore the relationship between urbanization and environmental quality [19,20]. The EKC hypothesis was first found by Krueger and Grossman in 1995, and has been widely applied to investigate the urbanization-environment system [21]. The EKC hypothesis shows that the relationship between urban development and environmental quality is an inverted U shape, meaning that environment quality first decreases in line with accelerated urbanization until an extreme point, after which it subsequently rises in correspondence with rising urbanization levels. Following Grossman and Kreuger, an increasing number of scholars have tested the EKC hypothesis using various methods and empirical analyses, with mixed results. Some researchers verified the accuracy of an inverted U-shaped curve through the EKC hypothesis [22,23]. However, other researchers argue that the EKC hypothesis may not be suitable for different research contexts, for example, for low-income countries [24,25,26,27].

In addition to mixed and inconsistent results, the Environmental Kuznets Curve (EKC) hypothesis is criticized for its theoretical shortcomings. The EKC hypothesis suggests that urbanization and the environment are independent of each other; this conclusion is highly restrictive [28]. In reality, the urbanization and environmental systems interact with and mutually restrict each other. Thus, the EKC hypothesis is inconsistent with the actual situation in real life. Coupling refers to the relationship between two or more systems which interact reciprocally through internal mechanisms [29]. The coupling theory originated in the field of physics, which in turn has recently been introduced into multidisciplinary research [30]. In recent years, a considerable number of researchers have started to pay attention to the relationship between urbanization and the eco-environment. The coupling theory [31,32], including resources utilization [33,34] and energy consumption [35,36], is used as the foundation of their research. For example, Hui et al. [37] investigated the coupling relationship between metropolitan scale expansion and land resource use intensity. Wang et al. [38] analyzed the coupling trend between urbanization and urban resources by building a static and dynamic coupling coordination degree evaluation model. Liu et al. [39] examined the coordination relationship between urbanization and CO_2_ emissions in Jinan City, also using the coupling theory. Thus, the coupling theory is a proven and effective method to examine the interaction relationship between eco-environments and urbanization systems.

Despite these advances, there is still a lack of understanding of the relationship between urbanization and the water ecosystem. Literature regarding the spatial and temporal analyses of the coordination coupling relationship between the two systems is limited. The majority of the existing studies focus on comparing the past and current coordination of a single region, without consideration of the comparison of the spatial gradients among different regions. As an important part of the ecological environment, the water ecosystem directly affects the sustainable development of society. China’s environmental issues related to its rapid pace of urbanization, and especially water ecological issues, are particularly prominent. Therefore, it is necessary to analyze the coordination between urbanization and the water ecosystem across spatial gradients and temporal scales to support sustainable urban development and water ecological management.

In the coupling coordination degree (CCD) model, it is important to select the contribution coefficients of two interactive systems. The coefficients reflect the contribution of the two systems to the overall coordination state [40]. In a traditional CCD model, the values of the contribution coefficient are determined through a subjective method. In most studies, the two coefficient values are arbitrarily assigned a value of 0.5 [41,42]. This method of subjectively assigning contribution coefficients has a clear disadvantage; the evaluation results will likely be inaccurate because they are influenced by human factors. As such, this method may not provide accurate insights for the coupling analysis between the water ecosystem and urbanization. To address this problem, this study introduces an improved CCD model for examining the coordination relationship between urbanization and the water ecosystem.

We attempt to analyze the coordination between urbanization and the water ecosystem in the Yangtze River Economic Belt (YREB) using the improved CCD model. We firstly develop a two-coupling analysis indicator system, which includes an urbanization indicator system and a water ecosystem indicator system. Secondly, we use the improved CCD model to examine and discuss the coupling relationship between urbanization and the regional water ecosystem across spatial gradients and temporal scales in the YREB. Finally, the obstacle degree model is utilized to diagnose the obstacle factors that hinder the coordination development between two systems. This study will provide a solid basis for the further development of sustainable urbanization practices.

## 2. Materials and Methods

### 2.1. Study Area

**Figure 1:**
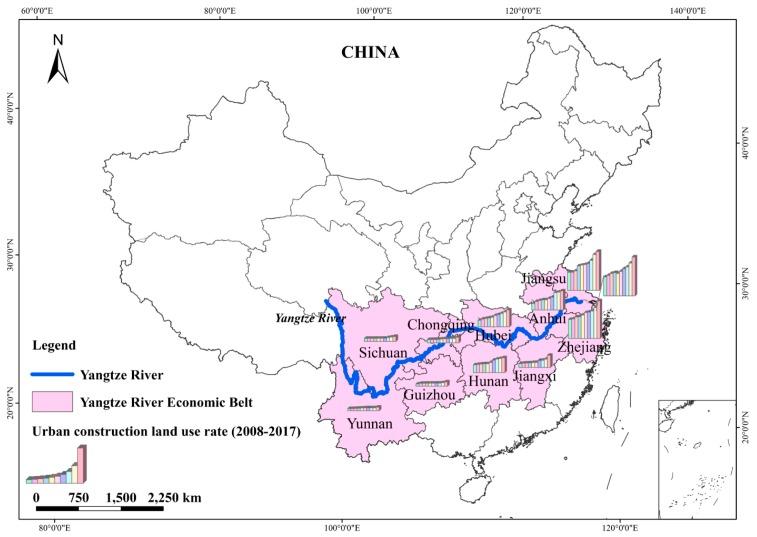
Yangtze River Economic Belt (YREB).

As the longest river in China, Yangtze River’s main stream flows through nine provinces and two municipalities. The area through which the Yangtze River flows is called the YREB. As the fastest growing urban agglomerations in China and a future high-density urbanization area [43], the YREB has become one of the most developed areas of China in the agriculture, industry, commerce, culture, education, science, and technology sectors. The YREB includes Jiangsu, Anhui, Zhejiang, Hubei, Jiangxi, Hunan, Sichuan, Yunnan, and Guizhou (nine provinces) and Chongqing and Shanghai (two municipalities) (see Figure 1). Its area is about 2.05 million square kilometers, corresponding approximately to 21% of the country’s total area. In 2016, the average level of urbanization was approximately 56.91%, while its total population and economy accounted for more than 40% of the national total [44]. Recently, the rapid development of urbanization has increased both the demand for water and the discharge of wastewater, leading to the deterioration of the water ecosystem. 

### 2.2. Methods

**Figure 2:**
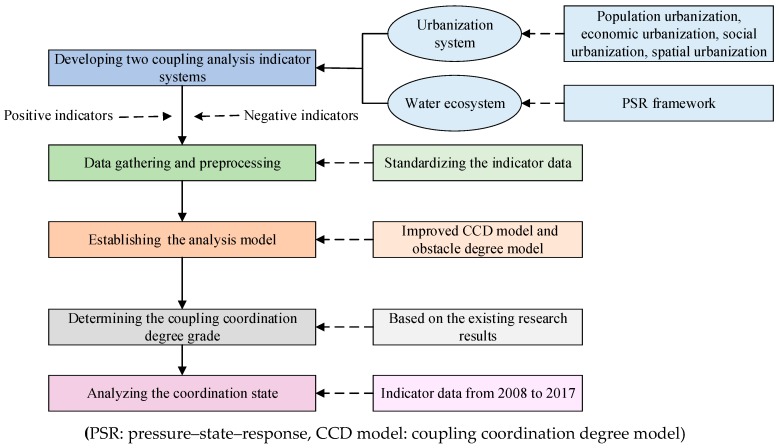
Framework of the methods in the coordination analysis among urbanization and water ecosystem (YREB, China).

In this paper, a systematic framework is developed as a means to examine the coordination relationship between water ecosystem and urbanization system (see Figure 2). The main analysis steps are: (1) design of the indicator system; (2) collection of indicator data and preprocessing; (3) establishment of the analysis model; and (4) analysis of the coupled coordination state.

#### 2.2.1. Indicator System Design

To explore the state of the water ecosystem in the urbanization process, it is necessary to establish two indicator systems, namely, an urbanization indicator system and a water ecosystem indicator system. The following four general criteria and principles are adopted in the selection of indicators [17,45,46]: (1) Pick the representative indicators. (2) Select the quantitative indicators required to facilitate the collection of indicator data. (3) Choose the comprehensive indicators in order to cover all components of urbanization and water ecosystem. (4) Follow the Chinese government’s policy documents or reports, such as the National New Urbanization Plan and the Water Ecological Comprehensive Governance Plan. 

**Figure 3:**
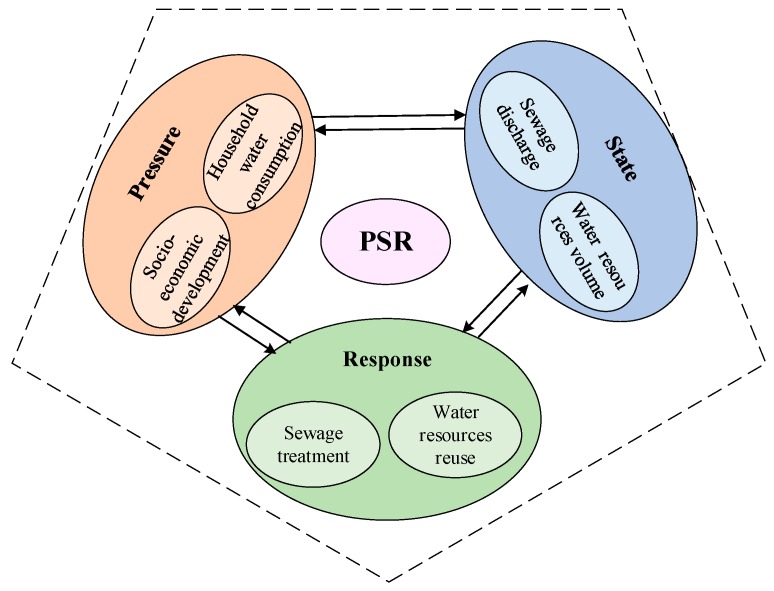
The PSR framework of reflecting the causes, consequences, and responses of the changes in the water ecosystem.

According to existing research related to new-type urbanization [17,18,19,47], we built an analysis indicator system of urbanization that covers population urbanization, economic urbanization, social urbanization, and spatial urbanization subsystems. The construction of the water ecosystem analysis indicator follows the pressure-state-response (PSR) framework, which is widely used in sustainable development research [48]. The PSR framework consists of three subsystems, namely, the pressure, state, and response subsystems. Of these three, the pressure subsystem can clearly reflect the pressure sources of the urban development on the water ecosystem. The state subsystem mainly represents the maintained state of the water ecosystem. The response subsystem illustrates a series of protection measures and response policies adopted by humans to protect the natural water ecosystem [49]. The water ecosystem’s PSR framework not only adequately displays the causal relationship between the three subsystems, but it also helps us understand the interrelationship between socio-economic development and the water environment (see Figure 3) [50]. In terms of the indicator layer, after fully considering the status quo of urbanization and socio-economic development in the YREB, we determined the urbanization indicator system through 14 representative evaluation indicators (see Table 1). In parallel, we chose 13 representative water ecosystem indicators, based on the state of the water ecosystem in the YREB (see Table 2).

#### 2.2.2. Data Sources and Preprocessing

The annual data on the indicators of urbanization system and water ecosystem were obtained from the China Statistical Yearbook (2008–2017) [51], the China Urban Statistical Yearbook (2008–2017) [52], the China Education Statistics Yearbook (2008–2017) [53], and the China Water Resources Bulletin (2008–2017), all of which are issued by the China Statistics Bureau [54]. 

The analysis indicators in the indicator system include both positive and negative indicators. With regard to the characteristics of the indicators, a higher positive indicator contributes to a better performance level, while a higher negative indicator contributes to a lower performance level. In order to unify the characteristics and scope of the indicators, it is necessary to standardize the selected indicators [55]. The normalization formulas are:

(1) $$\mathrm{Positive}\;\mathrm{indicator}:r_{ij}=(x_{ij}-\min\left\{x_{i}\right\})/(\max\left\{x_{i}\right\}-\min\left\{x_{i}\right\}),$$

and

(2) $$\mathrm{Negative}\;\mathrm{indicator}:r_{ij}=(\max\left\{x_{i}\right\}-x_{ij})/(\max\left\{x_{i}\right\}-\min\left\{x_{i}\right\}),$$

where $x_{ij}$ is the initial data of the indicator in *j* years, $r_{ij}$ is the normalized value of $x_{ij}$, i=1,2,⋯,m,j=1,2,⋯,n. 

#### 2.2.3. An Improved CCD Model 

The system that combines urbanization and water ecosystem can be regarded as a coupling system, in which urbanization and water ecosystem affect and restrict each other [56]. The coupling degree is a measure of the degree of correlation between systems. It is used to describe the degree of interactive influence between systems [57]. The improved CCD model presented in this study introduces the synergy theory as a means to determine the contribution coefficient values of the two systems. The synergy theory was first proposed by the famous physicist Haken in 1971 [58]. According to the synergy theory, mutual interaction and cooperation exists between different systems with different attributes. This theory is able to reveal the dynamic evolution process of coordination between several systems, from low to high levels. A high-level coordination degree between urbanization and water ecosystem can be achieved through a strong coupling between the two systems. This means that there is a strong coupling state and a small performance gap between these two systems [59]. Therefore, the steps of the improved CCD model are as follows [60]:

1. Calculation of the indicator weight 

The urbanization system and the water ecosystem cover 14 indicators and 13 indicators, respectively (see Table 1 and Table 2). The entropy method is used to distinguish the weight of the indicators [61,62]. The specific entropy weight calculation steps are proposed as follows:

(3) $$f_{ij}=r_{ij}/\sum_{j=1}^{n}r_{ij}$$

(4) $$u_{i}=-\frac{1}{\ln n}\sum_{j=1}^{n}f_{ij}\ln f_{ij}$$

(5) $$w_{i}=(1-u_{i})/(m-\sum_{i=1}^{m}u_{i})$$

where $f_{ij}$ is the proportion of the indicator *i*, *u_i_* is the entropy value, $w_{i}$ is the entropy weight.

2. Calculation of performance level

(6) $$U=\sum_{i=1}^{p}w_{i}^{u}\cdot r_{ij}^{u}$$

(7) $$W=\sum_{i=1}^{q}w_{i}^{w}\cdot r_{ij}^{w}$$

Here, *U* and *W* represent the performance level of the urbanization system and the performance level of the water ecosystem, respectively; *r_ij_^u^*, *w_i_^u^*, and *p* represent the normalized value, the weight value, and the number of indicators in the urbanization system, respectively. In addition, *r_ij_^w^*, *w_i_^w^*, and *q* represent the normalized value, the weight value, and the number of indicators in the water ecosystem, respectively.

3. Determination of improved contribution coefficients

(8) $$\alpha^{*}=W/(U+W)$$

(9) $$\beta^{*}=U/(U+W)$$

where $\alpha^{*}$ and $\beta^{*}$ are the improved contribution coefficients of urbanization system and water ecosystem, respectively. 

4. Determination of coupling coordination degree

(10) $$C={\left\{(U\cdot W)/{\left[(U+W)/2\right]}^{2}\right\}}^{1/2}$$

(11) $$T=\alpha^{*}U+\beta^{*}W$$

(12) $$D=\sqrt{C\cdot T}$$

where *C* refers to the coupling degree between the two systems, *T* refers to the comprehensive performance index of the two systems, and D is the degree of coupling coordination. 

The classification of the coupling coordination degree grade is the basis for analyzing the coordination relationship between water ecosystem and urbanization system. The coupling coordination degree *D* is divided into five grades in most studies, namely, low coupling, antagonism stage, running-in stage, coupling stage, and highly coupling. This is the basis for analyzing the coupling coordination state (Table 3) [39,63].

#### 2.2.4. Obstacle Factor Analysis 

In order to further explore the obstacle factors that affect the coordination state between the two systems, the obstacle degree model was used for analysis [64]. The analysis of obstacle factors can help to formulate and adjust new urban governance policy and water allocation measures in a targeted manner. The obstacle degree model is:

(13) Oi=Ii⋅wi(∑i=1mIi⋅wi)

Here, $I_{i}$ is the index skewness, $I_{i}=1-r_{ij}$; $O_{i}$ refers to the obstacle degree. The obstacle degree $O_{i}$ indicates the influence level of indicator i on the coupling coordination state between two systems. The larger the value of $O_{i}$ is, the greater the influence of factor i on the coupling coordination degree will be.

## 3. Results

### 3.1. Performance Level Trend Analysis

**Figure 4:**
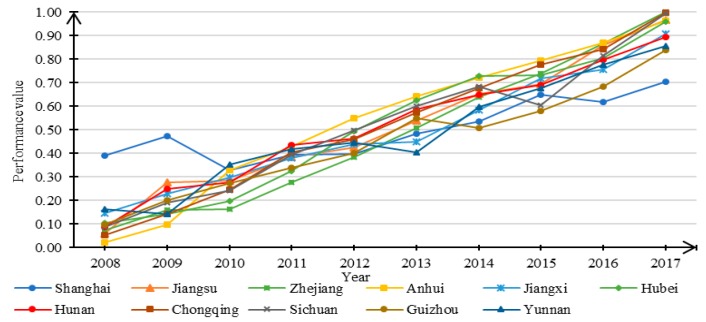
Trend in the performance levels of urbanization for 11 study regions from 2008 to 2017.

The performance values of urbanization system *U* can be obtained by applying Equation (6) and the data from the 11 regions. Figure 4 shows the trends of *U* in the YREB from 2008 to 2017. Figure 4 reveals an overall increasing trend in the urbanization performance levels across the investigated regions during the period from 2008 to 2017. In the past 10 years, except for a clear fluctuation in Shanghai City, the performance values of the other regions showed different degrees of increase. Anhui province had the highest growth trend, from 0.022 in 2008 to 0.963 in 2017. Shanghai had the smallest growth trend, with its highest level in 2008, while its lowest level occurred in 2017. This suggests that Anhui Province has experienced the fastest urbanization in the past decade, while Shanghai has had the smallest growth in terms of urbanization. It is noteworthy that the performance level of Chongqing has also shown significant growth; the performance value in 2017 was the highest among all investigated regions. Overall, in 2008, except for Shanghai, the other 10 regions remained at a low performance level. However, from 2010 onwards, all 11 regions maintained a relatively consistent upward trend.

**Figure 5:**
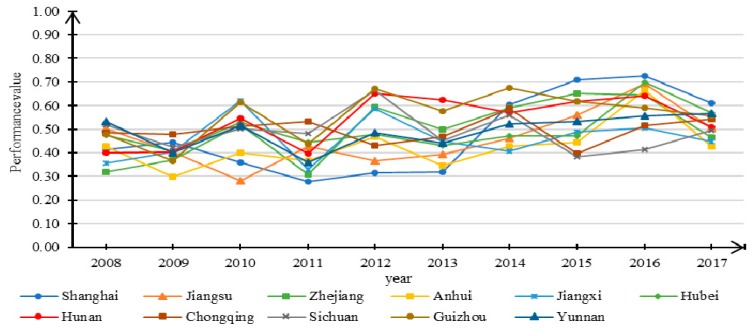
Trend in the performance levels of water ecosystem for 11 studied regions from 2008 to 2017.

By applying the data from the 11 regions in the YREB to Eq. (7), the performance values of water ecosystem *W* during the 2008–2017 period are obtained. The changing trend of *W* is shown in Figure 5. As shown in Figure 5, the rank order has a spindle-shaped distribution, with Shanghai and Jiangsu at the bottom, and Guizhou and Hunan at the top. In 2008, Zhejiang and Jiangxi had the worst water ecosystem performance, while Shanghai and Yunnan had the highest performance. In 2017, Shanghai’s performance level was the highest, while Anhui had the lowest. Overall, the performance level of the water ecosystem in the YREB followed a trend of slow growth. As shown in Figure 5, the performance level of the water ecosystem in Shanghai has experienced significant fluctuations, increasing initially before subsequently clearly decreasing from 2009 to 2011. Thereafter, the performance level rose from 2013 to 2016, and then considerably decreased in 2017. This fluctuation of the performance level of the water ecosystem is mainly attributed to the decline in the degree of health of the pressure and state subsystems in 2011 and 2017, respectively. Overall, the performance values of the water ecosystem in all 11 regions have different fluctuation trends during the period 2011 to 2017.

### 3.2. Spatial Characteristics Analysis 

**Figure 6:**
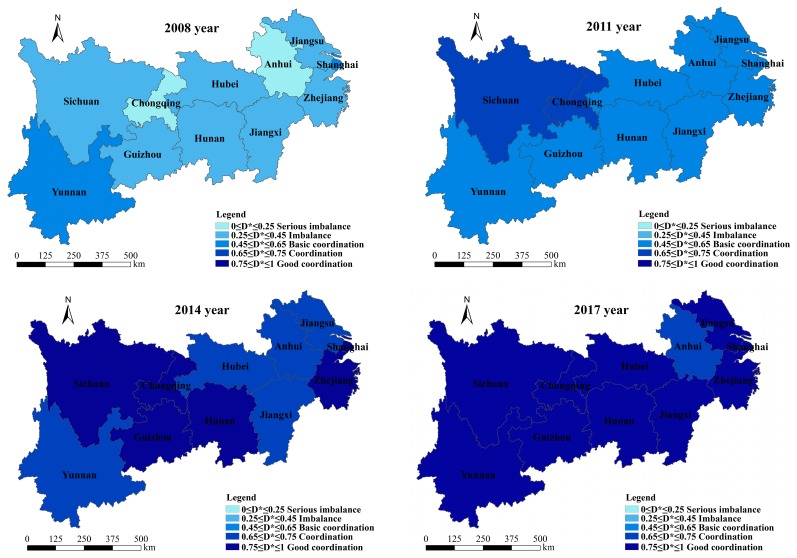
Spatial distribution of coordination state in the YREB.

The *D* values in the 11 regions are calculated for the period from 2008 to 2017. We divided the *D* into five coordination states, according to the classification standard of *D* values (Table 3); GIS software was used to visualize the spatial distribution of *D* across provinces and municipalities in the YREB from 2008 to 2017. The spatial distribution of the coordination state in the YREB is shown in Figure 6. Figure 6 shows that, in 2008, the *D* values among urbanization and water ecosystem in seven provinces were in an imbalanced state (i.e., Sichuan, Guizhou, Hubei, Hunan, Jiangxi, Jiangsu, and Zhejiang). Shanghai and Yunnan were in a state of basic coordination, while the two remaining regions (i.e., Anhui and Chongqing) were in a seriously imbalanced state. Moreover, in 2011, two regions (i.e., Sichuan and Chongqing) were in a state of coordination, while the remaining nine regions were in a state of basic coordination. In 2014, only six regions reached a good coordination state, while five regions were in a coordination state. Finally, in 2017, the coordination in all regions (except for Anhui Province) reached a state of good coordination. From 2008 to 2017, the *D* values in the central and eastern regions were lower than those in the western regions of the YREB. The coordination status between two systems in the west is better than that in east regions (as shown in Figure 6). 

### 3.3. Temporal Characteristics Analysis

**Figure 7:**
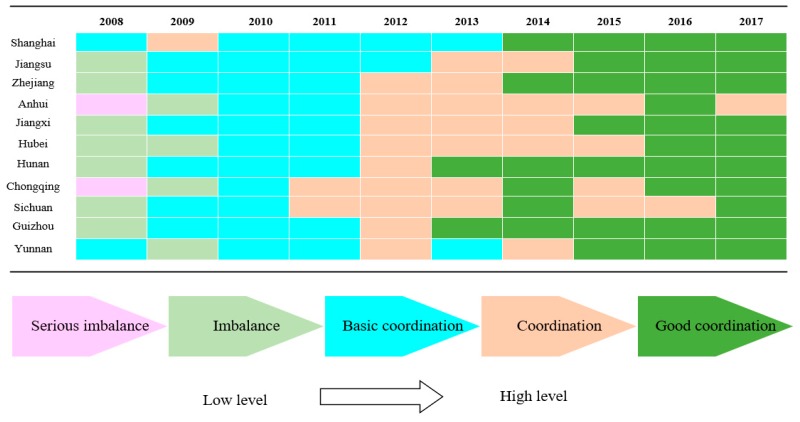
Trends in the coordination state in the 11 investigated regions from 2008 to 2017.

The trends of the coordination state between urbanization and the water ecosystem in all the YREB regions are shown in Figure 7. As shown in Figure 7, the coordination state between the two systems followed a gradually improving trend, moving from the serious imbalance in 2008 to one of good coordination in 2017. A change in the coupling system seemed to occur in three different three-year periods (2008–2011, 2011–2014, and 2014–2017). Our analysis took these periods into account, examining the coordination state, the urbanization performance trend, and the water ecosystem performance trend. The development of the coupling coordination over the three periods can be described as follows:

(1) 2008–2011: The relationship between the two systems was initially at a low-coordination level, but this improved rapidly. In 2008, the two systems showed an imbalanced state in all regions, except for Shanghai and Yunnan. As the level of urbanization increased rapidly in 2009, the performance of the water ecosystem decreased, due to the impact of urbanization. From 2009 to 2011, the *D* values between the two systems grew more rapidly; the gap between the *D* values of each region constantly narrowed. Therefore, the coordination state in the YREB was constantly optimized. In 2011, the influence of urbanization on the water ecosystem weakened, and the quality of the water ecosystem was still within a recoverable range. As a result, the two systems reached a basic coordination state in all of the 11 analyzed regions. 

(2) 2011–2014: The link between the two systems began to be reinforced, and the level of coordination between the two systems improved steadily. During this period, the performance levels of both the urbanization system and the water ecosystem gradually increased; the coordination state between the two systems improved from a state of “basic coordination” to a state of “coordination”. In synthesis, in 2014, the urbanization and water ecosystem in all regions of the YREB were in a coordination state.

(3) 2014–2017: The coordination state between the two systems slowly improved. After the launch of the “Belt and Road” initiative in 2013, the urbanization of the YREB experienced a period of increasing development. In addition, a series of water ecological protection measures were proposed during this period, such as the rain and sewage diversion, sewage treatment, and the construction of “Sponge City”, all of which improved to some extent the quality of the YREB water ecosystem. Both the level of urbanization and the quality of the water ecosystem continued to increase during this period. However, the water ecosystem performance level was not synchronized with the urbanization performance level. In fact, the growth of the water ecosystem was relatively stable, while urbanization experienced rapid growth. Therefore, the coordination relationship between the two systems improved slowly. In short, in 2017, the coordination between the urbanization and water ecosystem in the YREB was good.

### 3.4. Obstacle Factors Analysis

The obstacles degree of each subsystem in the urbanization and water ecosystem are shown in Table 4. By referring to Table 4, we can find the main subsystems of urbanization system and water ecosystem that restrict the coordinated development of both systems in the 11 investigated regions. Specifically, in the urbanization system (except for Chongqing), the spatial urbanization subsystem represented the greatest obstacle in terms of degree, whereas population urbanization had the lowest degree. This means that spatial urbanization was the main subsystem obstacle in these 10 regions, while population urbanization was the least important subsystem. In Chongqing City, social urbanization was the key subsystem in terms of hindering the development of the coordination state between the two systems. In the water ecosystem, the pressure subsystem in 10 regions (except in Hubei Province) represented the biggest obstacle by degree, while the response subsystem had the smallest. This shows that in the water ecosystem, pressure was the key subsystem affecting the coordination state between urbanization and water ecosystem in the 11 investigated regions. By comparison, the state subsystem had a minimal impact on the state of the water ecosystem in the YREB.

Table 5 lists the main obstacle indicators in the urbanization system and water ecosystem. As shown in Table 5, the main obstacle indicators affecting the coordinated development of the two systems in the 11 studied regions were distributed throughout different subsystems. In the urbanization system, the indicators of *U_2_* (population growth rate), *U_11_* (urban population density), and *U_12_* (urban road area per capita) ranked as the top three indicators in all regions. They came from the population urbanization and spatial urbanization subsystems. This means that these three indicators are considered to exert the greatest influence on the urbanization system in the YREB. As a representative example of China’s rapid urban development, the YREB had experienced rapid urban population growth over the past decade, which in turn led to an increase in population density and a reduction in per capita infrastructure. This is the main hindrance to the coordinated development of the two systems. In the water ecosystem, the indicators of *C_1_* (water consumption per unit of GDP), *C_2_* (water consumption of industrial output), and *C_6_* (total volume of water resources) were the top three obstacle indicators in all regions. This implies that these three indicators had the greatest impact on the YREB water ecosystem. All three indicators were from the pressure subsystem and state subsystem, and this finding is consistent with the obstacle degree subsystem analysis.

## 4. Discussion

The improved CCD model was used to analyze the coordination relationship between urbanization and the water ecosystem in the YREB. The performance level results show that the urbanization performance values experienced a clear upward trend across the 11 investigated regions during the 2008–2017 period. In the 11 regions, Anhui Province experienced the most significant increase in terms of performance level, while Shanghai had the smallest increase. Regarding the water ecosystem, the performance values in each region showed a fluctuating increasing trend. However, compared with the urbanization system, the overall growth trend of the water ecosystem is not clear. Overall, the YREB has experienced rapid urbanization, whereas the performance level of water ecosystems has not improved in the past decade. According to this finding, policy makers should pay closer attention to protecting the water environment when drafting any new-urbanization development plans.

According to the results of the *D*, the spatial characteristics and temporal evolution trends of the coordination state between urbanization and the water ecosystem in the YREB can be analyzed. From a spatial point of view, the coordination state between the two systems in the YREB gradually increased from east to west. This was mainly due to the rapid industrialization and urbanization in the central and eastern regions, which mainly rely on industries that create significant amounts of pollution and consume significant amounts of water. In addition, the area’s water ecological protection has been neglected in terms of the utilization of water resources and in the rapid urbanization process. This failure has caused serious water pollution and led to a low level of water ecosystem performance, which in turn caused the interaction between the two systems to weaken. Compared with the central and eastern regions, the industrial development in the western region is backward and the urbanization process is slow. There are fewer enterprises that have high water consumption levels and cause significant pollution. As such, the water ecosystem in this region is in a better state. Thus, the coordination between the two systems in the western region is strong.

In terms of the temporal scale, the coordination state between the two systems experienced an initial period of imbalance or antagonism, coupled with rapid growth (2008–2011). This was followed by a period of basic coordination with steady growth (2011–2014), and finally a period of good coordination with slow growth (2014–2017). Figure 7 shows that the coupling coordination level between the two systems in each region evolved during the study period from a state of serious imbalance to a state of good coordination. This indicates that the coordination state between urbanization and the water ecosystem in the YREB was gradually optimized and continuously improved with the passage of time. From 2008 to 2011, the coordination state between the two systems evolved from an imbalanced state to a state of basic coordination. From 2011 to 2014, the nexus between the two systems continued to be reinforced, and the level of coordination between the two systems steadily improved. After 2014, corresponding water environmental governance measures and urbanization development policies were proposed; the performance level of both systems was thereby improved. Therefore, in 2017, the coordination state between the two systems basically reached one of good coordination in all 11 regions. Overall, with the passage of time, the coordination state of each region gradually improved, and the spatial differences between different regions gradually narrowed.

The obstacle degree analysis results show that spatial urbanization and pressure are the two main obstacle subsystems. Meanwhile, the population urbanization and response subsystems had the least influence on the degree of coordination between the two systems in the YREB. These findings are consistent with the status quo. They suggest that, in the development of urbanization in the YREB, the urban spatial scope has continued to expand, and the pressure on the natural water ecosystem is increasing. The continuous expansion of spatial scope and the increasing pressure on the water ecosystem are the main factors hindering the coordinated development between the two systems. The YREB is undergoing rapid economic and industrial development, and the demand for water resources is increasing sharply. In view of these circumstances, more effective water resource protection measures are needed in order to reduce the pressure on water resources and to improve the state of the water ecosystem.

## 5. Conclusions

The YREB is an important part of China’s One Belt and One Road strategy. Hence, it is important to analyze the coordination between urbanization and the water ecosystem for the sustainable development of the region. To this purpose, this study established two indicator systems for urbanization and the water ecosystem. Then, an improved CCD model was used to examine the coordination between the two systems in the YREB from 2008 to 2017, as well as the differences in coordination between spatial gradients and at different temporal scales. Finally, the main obstacle subsystems and indicators were diagnosed using the obstacle degree model. The main findings are as follows.

First, the urbanization performance values of all regions, except for Shanghai, from 2008 to 2017 were shown to follow an upward trend, though with some important differences, while Shanghai was shown to follow a clear fluctuation trend. Anhui and Chongqing had the most evident urbanization growth, while Shanghai had the least significant growth. In terms of water ecosystem performance, the performance values of the water ecosystem in each province and municipality were shown to follow a fluctuating increasing trend. However, the overall growth trend was not evident, and Yunnan and Chongqing had the smallest growth trend.

Second, the *D* values between urbanization and the water ecosystem followed an overall increasing trend from 2008 to 2017. The coordination between the two systems in the YREB experienced a dynamic evolution, from a serious imbalance toward a good coordination state. From a spatial perspective, the coordination state between the two systems gradually increased from east to west; moreover, the spatial difference across regions gradually narrowed with the passage of time. In terms of temporal scale, the coordination between the two systems from 2008 to 2017 experienced an evolution from a serious imbalance to a good coordination state, passing from an initial period of imbalance or antagonism with rapid growth (2008–2011), through a period of basic coordination with steady growth (2011–2014), toward a period of good coordination with slow growth (2014–2017).

Third, regarding the obstacle degree analysis, spatial urbanization and pressure are the main obstacle subsystems, while the population urbanization and response subsystems have had the least influence on the degree of coordination between the two systems in the YREB. In the indicator layers, the urbanization indicators of *U_2_* (population growth rate), *U_11_* (urban population density), and *U_12_* (urban road area per capita rank), as well as the water ecosystem indicators of *C_1_* (water consumption per unit of GDP), *C_2_* (water consumption of industrial output), and *C_6_* (total volume of water resources) are considered to be the main factors hindering the coordinated development of urbanization and the water ecosystem. 

According to these findings, the government should pay closer attention to spatial urbanization when drafting any new-urbanization development plans. Environmental considerations are becoming increasingly important in the process of sustainable urban development. The government can increase investment in industrial water pollution control in order to control pollution emissions. Water utilization efficiency can also be improved in order to reduce water resource stress. In order to ensure the harmonious development of the water ecosystem and urbanization, the national government should develop an institutional guarantee system to ensure the balanced development of urbanization and the protection of the water ecosystem. The government should also formulate different economic and environmental development plans, based on the degree of coupling coordination in different regions. In addition, given the imbalance in the harmonious development between different areas, local governments should strengthen their cooperation and exchanges with adjacent regions. They should also share resources, thus achieving a win–win situation.

Given the fact that our study is intended to explore the coordination relationship between urbanization and the water ecosystem in China’s YREB, the design of the indicator systems is based on the comprehensive consideration of the urban development status and the water ecosystem situation in the YREB. Thus, our indicator systems may not be applicable to other countries and regions. In addition, the classification standard of the coordination state may differ, given the different regions and periods. It is recommended that future research should update the indicator systems and the coordination state criteria to meet the needs of various regions and periods.

## Figures and Tables

**Table 1:** Indicator system for the urbanization system.

Subsystem	Indicators	Unit	Nature
Population urbanization	*U_1_* Population urbanization rate	%	+
	*U_2_* Population growth rate	%	+
Economic urbanization	*U_3_* GDP per capita	Yuan	+
	*U_4_* Per capita disposable income	Yuan	+
	*U_5_* Percentage of GDP of tertiary industry	%	+
	*U_6_* Per capita investment in fixed assets	10^4^Yuan	+
Social urbanization	*U_7_* Dwelling area per capita	m^2^/person	+
	*U_8_* Per capita education funds	10^4^Yuan	+
	*U_9_* Number of college students	-	+
	*U_10_* Number of public transportation vehicles	-	+
Spatial urbanization	*U_11_* Urban population density	Persons/km^2^	−
	*U_12_* Urban road area per capita	km2/person	+
	*U_13_* Percentage of built-up areas in total land area	%	+
	*U_14_* Built-up area per capita	km2/person	+

**Table 2:** Indicator system for the water ecosystem.

Subsystem	Indicators	Unit	Nature
Pressure	*C_1_* Water consumption per unit of GDP	10^9^m^3^	−
	*C_2_* Water consumption of industrial output	10^9^m^3^	−
	*C_3_* Water consumption of agricultural irrigation	10^9^m^3^	−
	*C_4_* Household water consumption	10^9^m^3^	−
	*C_5_* Urban sewage discharge	10^9^m^3^	−
State	*C_6_* Total volume of water resources	10^9^m^3^	+
	*C_7_* Per capita water occupancy volume	m^3^	+
	*C_8_* Per capita water consumption	m^3^	+
	*C_9_* Water production coefficient	%	+
Response	*C_10_* Industrial water pollution control investment	10^4^ Yuan	+
	*C_11_* Urban sewage treatment rate	%	+
	*C_12_* Urban water reuse rate	%	+
	*C_13_* Urban water supply rate	%	+

**Table 3:** Classification standard and the types of coupling coordination degree.

Coordination State	*D* Value	Coupling Level	Description
Serious imbalance	0 ≤ *D* ≤ 0.25	Low coupling	The nexus between the two systems is very poor.
Imbalance	0.25 < *D* ≤ 0.45	Antagonism stage	The interaction between the two systems is weak.
Basic coordination	0.45 < *D* ≤ 0.65	Running-in stage	The link between the two systems begins to reinforce.
Coordination	0.65 < *D* ≤ 0.75	Coupling stage	The relationship between the two systems is coordinated.
Good coordination	0.75 < *D* ≤ 1	Highly coupling	The coordination between the two systems is very good.

**Table 4:** Obstacle degree of subsystem in urbanization and water ecosystem.

System	Urbanization				Water Ecosystem		
Subsystem	Population Urbanization	Economic Urbanization	Social Urbanization	Spatial Urbanization	Pressure	State	Response
Shanghai	0.168	0.285	0.227	0.320	0.366	0.361	0.273
Jiangsu	0.166	0.274	0.219	0.340	0.378	0.372	0.300
Zhejiang	0.149	0.274	0.210	0.368	0.356	0.322	0.322
Anhui	0.207	0.290	0.225	0.278	0.357	0.340	0.302
Jiangxi	0.186	0.239	0.272	0.302	0.353	0.325	0.322
Hubei	0.142	0.267	0.246	0.346	0.283	0.369	0.348
Hunan	0.126	0.270	0.271	0.333	0.385	0.341	0.274
Chongqing	0.193	0.256	0.308	0.243	0.372	0.324	0.304
Sichuan	0.191	0.241	0.240	0.329	0.351	0.338	0.311
Guizhou	0.154	0.267	0.251	0.328	0.367	0.330	0.323
Yunnan	0.135	0.258	0.243	0.364	0.369	0.322	0.309

**Table 5:** The main obstacle indicators in urbanization and water ecosystem.

Urbanization						Water Ecosystem				
Indicator Order	1	2	3	4	5	1	2	3	4	5
Shanghai	*U12*	*U2*	*U11*	*U6*	*U4*	*C1*	*C2*	*C6*	*C4*	*C5*
Jiangsu	*U12*	*U2*	*U11*	*U6*	*U14*	*C1*	*C6*	*C2*	*C3*	*C7*
Zhejiang	*U12*	*U2*	*U11*	*U14*	*U4*	*C1*	*C2*	*C6*	*C8*	*C4*
Anhui	*U2*	*U12*	*U11*	*U14*	*U6*	*C1*	*C6*	*C2*	*C3*	*C9*
Jiangxi	*U2*	*U12*	*U11*	*U10*	*U14*	*C1*	*C2*	*C6*	*C12*	*C4*
Hubei	*U12*	*U2*	*U11*	*U4*	*U6*	*C1*	*C2*	*C6*	*C7*	*C12*
Hunan	*U12*	*U12*	*C11*	*C10*	*U4*	*C1*	*C2*	*C6*	*C8*	*C4*
Chongqing	*U12*	*U2*	*U11*	*U6*	*U4*	*C1*	*C6*	*C2*	*C8*	*C7*
Sichuan	*U2*	*U12*	*U11*	*U14*	*U6*	*C1*	*C6*	*C2*	*C3*	*C12*
Guizhou	*U2*	*U12*	*U11*	*U13*	*U6*	*C1*	*C2*	*C6*	*C4*	*C10*
Yunnan	*U12*	*U2*	*U11*	*U13*	*U4*	*C1*	*C2*	*C6*	*C7*	*C5*
